# EHMTI-0014. A case of ice-pick headache

**DOI:** 10.1186/1129-2377-15-S1-D64

**Published:** 2014-09-18

**Authors:** D Tertan, OB Tertan

**Affiliations:** 1 Neurology, Clinical Hospital Pelican, Oradea, Romania; 2 University of Medicine and Pharmacy, Cluj Napoca, Romania

## Introduction

Ice-pick pain or ophtalmodynia periodica consists of unilateral, ultrashort and localized stabs of pain in the distribution of the first branch of the trigeminal nerve and occurs in the absence of organic disease.

## Aim

To draw attention to the possibility of coexistence of a chronic migraine with a stabbing pain in a pregnant patient.

## Methods

This case involves a female, aged 39, pregnant -12 weeks, experiencing stabbing light to 15-20 times per day on average. Before pregnancy, her pains were completely relieved with Indomethacin 25 mg three times daily for seven days and 6 mg of melatonin per day at bedtime: while continuing to take melatonin, she remained pain-free at a two-month-follow-up. Because these drugs were contraindicated in her status, we used Capsaicin nasal spray which improved the reccurent ice-pick pains-4-5 attacks per day, but with extreme headache-related disability. We suggested a preventive treatment with Boswellia serrata 350-three times a day which stopped completely the reccurent ice-pick pains for the entire period of pregnancy.

## Results

In this particular case, Melatonin and Indomethacin, being contraindicated, a herbal treatment was appropriate and useful. Boswellic acid inhibits prostaglandin synthesis by inhibiting the lipoxygenase pathway.

## Conclusion

Physicians need to be knowledgeable about this syndrome because each has its own treatment and the patient can be burdened with excruciating headache-related disability. Boswellia might be an effective option for it.

No conflict of interest.

